# Onset and Progression of Behavioral and Molecular Phenotypes in a Novel Congenic R6/2 Line Exhibiting Intergenerational CAG Repeat Stability

**DOI:** 10.1371/journal.pone.0028409

**Published:** 2011-12-07

**Authors:** Randi-Michelle Cowin, Nghiem Bui, Deanna Graham, Jennie R. Green, Stephan Grueninger, Lisa A. Yuva-Paylor, Arsalan U. Syed, Andreas Weiss, Richard Paylor

**Affiliations:** 1 Department of Molecular and Human Genetics, Baylor College of Medicine, Houston, Texas, United States of America; 2 Novartis Institutes for BioMedical Research, Neuroscience Discovery, Basel, Switzerland; 3 Department of Neuroscience, Baylor College of Medicine, Houston, Texas, United States of America; Louisiana State University Health Sciences Center, United States of America

## Abstract

In the present study we report on the use of speed congenics to generate a C57BL/6J congenic line of HD-model R6/2 mice carrying 110 CAG repeats, which uniquely exhibits minimal intergenerational instability. We also report the first identification of the R6/2 transgene insertion site. The relatively stable line of 110 CAG R6/2 mice was characterized for the onset of behavioral impairments in motor, cognitive and psychiatric-related phenotypes as well as the progression of disease-related impairments from 4 to 10 weeks of age. 110Q mice exhibited many of the phenotypes commonly associated with the R6/2 model including reduced activity and impairments in rotarod performance. The onset of many of the phenotypes occurred around 6 weeks and was progressive across age. In addition, some phenotypes were observed in mice as early as 4 weeks of age. The present study also reports the onset and progression of changes in several molecular phenotypes in the novel R6/2 mice and the association of these changes with behavioral symptom onset and progression. Data from TR-FRET suggest an association of mutant protein state changes (soluble versus aggregated) in disease onset and progression.

## Introduction

Huntington's disease (HD) is a progressive neurodegenerative disorder characterized by a variety of motor, cognitive, and psychiatric symptoms. Caused by an expansion of the polyglutamine (Q) tract in the human Huntington's disease gene, *HTT*, HD occurs in patients with greater than 35 CAG repeats, while expansions in the range of 80–100Q results in the juvenile form of the disease [1], [2], [3], [4]. The R6/2 line is one of the most frequently studied mouse models of HD due to the relatively early onset and severity of disease phenotypes [5]. To date most of the R6/2 studies have used transgenic mice from a mixed CBA.C57BL/6 (CBA.B6) genetic background. It is well known that genetic background can have dramatic effects on a number of behavioral phenotypes [6], [7], [8], therefore, it is also important to create and study R6/2 mice on a more controlled genetic background.

Recently, Menalled et al. [6] reported the first results of the behavioral phenotypes of R6/2 mice on a congenic C57BL/6J (B6) genetic background; however, the transgenic mice from this study carried approximately 260 CAG repeats, a CAG repeat length that is considerably longer than is commonly seen in human patients. In addition, mice carrying CAG repeat numbers in this range or larger have recently been shown to exhibit variable phenotypes [6], [9], [10]. The goal of the present study was to develop a congenic line of R6/2 transgenic mice that contained a CAG repeat length in a range that is more comparable to other mouse models of HD (e.g. R6/1, N171-82Q, YAC72, YAC128 and many of the knock-in lines) [5], [11], [12], [13], [14], [15], [16], [17]. Using speed congenics we generated a line of R6/2 transgenic mice that express 110–120 CAG on a pure B6 genetic background and characterized the onset and progression of a number of behavioral phenotypes in this new B6 110Q R6/2 line. In addition, we examined a number of molecular markers including mRNA expression and newly developed FRET-based protein assessments of both soluble and aggregated HTT protein [18]. Although a potentially important and revealing aspect of diseased progression and characterization, there have been no previous studies reporting a formal investigation of the molecular changes that occur concurrently with decline in behavior phenotypes of in R6/2 mice.

The findings from the present study indicate that behavioral phenotypes in this B6 110Q R6/2 line emerge and progress from 4 to 10 weeks of age and that these changes are associated with concurrent changes in soluble and aggregated protein levels, but not transgene transcript expression. In addition, quantification of CAG repeat length indicates that this particular line of R6/2 mice has a minimal level of intergenerational instability. Finally, because future studies from our lab will be directed at using this new line to identify potential modifiers of HD, we report our characterization of the original R6/2 insertion site [5] on mouse chromosome 4. To our knowledge, this is the first study to identify the location of the R6/2 transgene. We believe the 110Q R6/2 line described in the present study offers a number of features that will be useful to investigators interested in using R6/2 mice to better understand HD.

## Results

### CAG Repeat Stability

The R6/2 line of mice was created by a random insertion of a construct containing the first exon of the human *HTT* gene, driven by the endogenous promoter [5]. While originally generated expressing approximately 150 CAG repeats [5], the CBA.B6 R6/2 mice obtained for the current study from Jackson Laboratories, descendant from the mouse line originally characterized by Mangiarini et al. [5], carried a contracted repeat length of approximately 110 polyglutamine (Q) (Table 1).

**Table 1:** Average repeat length of three R6/2 lines showing intergenerational stability or instability and the minimum and maximum repeat lengths identified in each generation.

	110Q			Line X			Line Y		
Generation	Mean ± SEM	Min	Max	Mean ± SEM	Min	Max	Mean ± SEM	Min	Max
Received	108.9±0.59	101	118	249.7±2.60	235	260	286.8±1.90	278	291
F1	110.6±0.70	101	119	260.3±1.60	231	275	291.3±1.60	278	304
N2	110.8±0.20	109	114	249.8±0.70	246	254	302.3±1.50	272	329
N3	111.2±0.20	109	113	252.1±1.60	231	263	309.2±1.50	287	324
N4	112.7±0.80	110	116	255.1±7.20	183	279	313.1±1.90	256	326
N5	110.9±0.30	108	112	281.0±1.80	265	295	333.6±3.10	304	469
N6	109.8±0.40	108	112				336.7±4.20	304	398
N7	109.9±0.20	107	112				344.1±4.40	315	359
N8	110.8±0.30	108	114						
N9	111.2±0.24	109	114						
N10	112.3±0.15	109	115						
N11	112.5±0.25	110	116						
N12	112.4±0.50	101	115						
N13	114.6±0.50	112	117						
Average changeper generation	.438 CAG			6.260 CAG			8.186 CAG		

**Figure 1:**
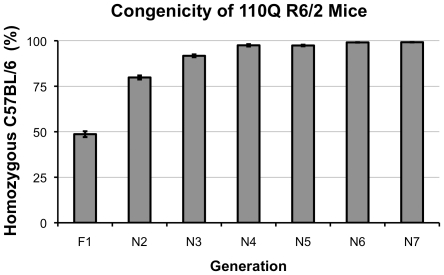
Congenicity across multiple generations of backcrossing R6/2 mice to C57BL/6J. Average percentage of homozygous B6 markers based on the 96-marker panel. Average percent B6 homozygosity was less than 50% after the first backcross but reached congenicity of 95% by the N4 generation.

In order to efficiently create a pure and congenic B6 R6/2 line we employed a speed-congenics breeding scheme. Briefly, a panel of 96 SSLP (single nucleotide length polymorphism) markers was selected throughout the genome for PCR genotyping. In each generation, male progeny were genotyped both for the presence of the exon 1 transgene as well as for the allele type (CBA or C57B/6J) for each of the 96 makers selected. Mice with the highest percentage of B6 alleles were bred preferentially. Although DNA from original mice received from Jackson Laboratories, Inc. were not genotyped with the SSLP panel, we found that after the first backcross to pure B6, 110Q mice in our colony ranged from 39%–61% homozygous B6 for the selected markers. The selective breeding technique resulted in a line of congenic R6/2 mice as early as 4 generations (95%–100% homozygous C57BL/6) with the entire population 99%–100% congenic (as defined by the SSLP marker panel) within 7 generations (Figure 1).

Early in the process of backcrossing the 110Q R6/2 mice, however, it became apparent that our line of 110Q R6/2 mice exhibited a distinct trait. Namely, the intergenerational repeat expansions commonly observed in R6/2 mice [6], [9], [10], [19] did not occur with the expected progressive expansion rate in our congenic line (Table 1). In fact, the variability of repeat length within a generation appeared to decrease with successive generations. We also measured the repeat length across multiple generations of breeding in two other R6/2 lines bred within our colony at Baylor College of Medicine as a comparison with other B6 congenic R62 lines with longer CAG repeats [6], [9], [10]. We found that in the other lines of R6/2 mice bred within our colony, CAG repeat lengths readily expanded across 6–8 generations (Table 1). Therefore, the lack of intergenerational expansion observed in this new B6 110Q R6/2 line is not the result of something unique to our breeding program.

### Characterization of the Transgene Insertion Site

**Figure 2:**
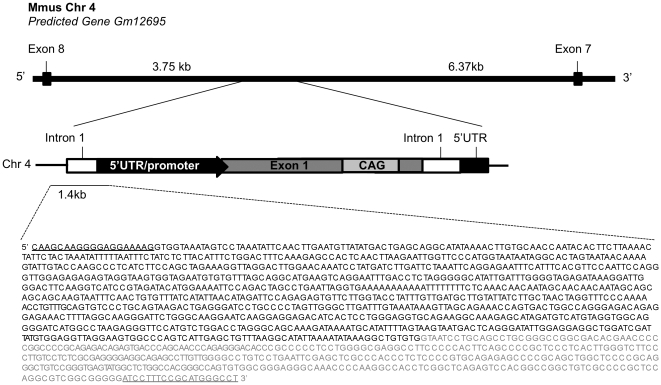
Location of the insertion site of the R6/2 transgene. The insertion site localized to a position on mouse chromosome 4 in an intron of predicted gene GM12695. The junction of the 5′ insertion site was sequenced through approximately 1400 bp of the transgene. The represented structure of the R6/2 insertion (for areas not sequenced) is based on the report by Mangiarini et al. (1996). Underlined sequences represent the primers used to amplify across the junction. Sequence given in black represents chromosome 4. Sequence given in grey represents transgene.

During the creation of the congenic 110Q line, 1 of the 96 SSLP markers chosen for the speed congenics panel was found to be extremely resistant to homologous recombination and remained heterozygous for many generations after successfully obtaining congenicity in the other 95 markers. We interpreted the difficulty of recombining this SSLP marker to the B6 allele as suggesting that the random and unnamed insertion site of the R6/2 transgene was near the chromosomal location of the marker D4Mit303 located on mouse chromosome 4: 97876919–97877039 (NCBI Build 37.2). To confirm that the transgene had integrated near the D4Mit303, PCR-based GenomeWalker technology was used to isolate genomic sequences flanking the transgene insertion site. Sequence analysis of the junction indentified the insertion site as within an intron of the predicted gene Gm12695 on chromosome 4. Relative to the forward strand, the exact insertion site is 3′ of base-pair 4: 96409480 (assembly NCBI 37/mus musuculus 9) (Figure 2). Because this line of mice is a direct descendant of the original 150Q R6/2 line [5], we expect the identification of this chromosomal location as the insertion site would be confirmed in 150Q mice.

### Behavioral Characterization

In order to identify the age of onset and pattern of disease progression in the minimally unstable and congenic 110Q R6/2 mice, behavioral analysis was performed. Male and female 4, 6, 8, and 10 week old R6/2 and wild-type mice were tested in a variety of tests to assay for a number of domains of CNS function including motor coordination, exploratory activity, anxiety-related responses, sensorimotor gating, and learning and memory.

#### R6/2 mice show reduced activity and exploration in the open field assay at an early age

**Figure 3:**
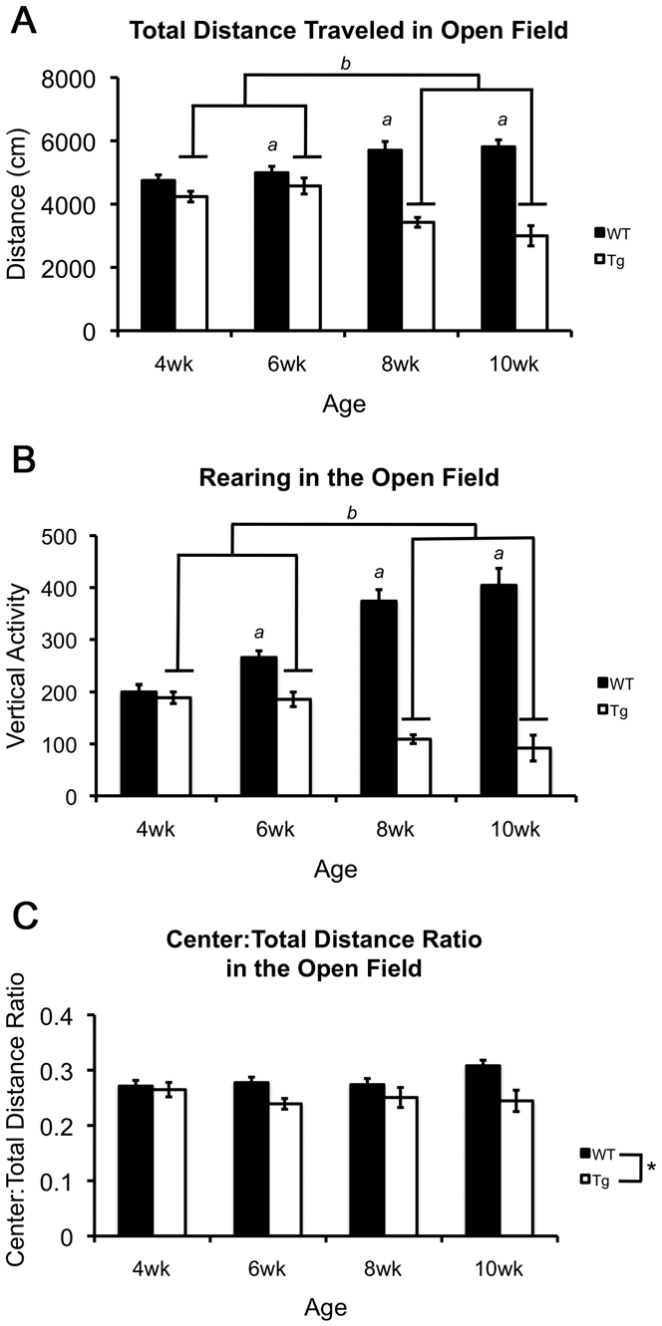
Behavioral responses of B6 R6/2 transgenic and wildtype mice across 4 ages in the open-field test. (**A**) Exploratory activity as measured by total distance traveled in an open-field. (**B**) Rearing behavior as measured by vertical activity (beam interruptions). In both **A** and **B**, ‘*a*’ represents that R6/2 mice are significantly less active than wildtype littermates at 6, 8 and 10 weeks of age. ‘*b*’ represents that 8 and 10 week old R6/2 mice are less active than 4 and 6 week old R6/2 mice. Wildtype mice increase in activity from 6 to 8 weeks of age. (**C**) Anxiety-like responses as measured by the center∶total distance ratio. ‘*’ represents that overall B6 R6/2 transgenic mice spend less of their exploration in the center of the open-field relative to wildtype mice. All p-values≤0.022 by three-way ANOVA and simple effects analysis.

R6/2 and wild-type mice were tested in the open field assay to assess disease onset and progression in activity and exploration. Overall, transgenic mice had significantly lower activity measures of total distance than wild-type littermates [F(1,203) = 98.757, p<0.001]. However, a significant genotype×age interaction was found [F(1,203) = 16.256, p<0.001] indicating that the genotype effect was age dependent. Specifically, transgenic mice explored less than wildtype animals at 8 and 10 weeks of age. Additionally, transgenic 110Q mice exhibited similar levels of exploration at 4 and 6 weeks of age, but at 8 and 10 weeks of age the activity of transgenic mice was significantly reduced (p-values<0.001) (Figure 3a). Within wildtype mice, activity was significantly increased in 8 and 10 week old mice relative to 4 and 6 week old wildtypes (p-values≤0.008).

Similar to the exploratory activity, rearing behavior in the open-field was also decreased overall in R6/2 transgenic mice compared to wild-type littermates [F(1,203) = 176.273, p<0.001], but this difference was age dependent as indicated by a genotype×age interaction [F(1,203) = 29.819, p<0.001]. Simple effects analysis revealed that transgenic mice rear significantly less than controls at 6, 8 and 10 weeks of age (p-values≤0.001), but not at 4 weeks. Changes across age were also identified in both genotypes. Specifically, transgenic mice reared less at 8 and 10 weeks than mice at 4 and 6 weeks of age (p-values≤0.001). In wildtype mice, rearing increased significantly from 4 to 6 and 6 to 8 weeks of age (p-values≤0.022) (Figure 3b).

#### Anxiety measures in the open field are present in transgenic animals at specific ages

To assess anxiety-like behavior in the open field, the distance traveled in the center of the open field is divided by the total distance traveled in the entire field (center∶total distance ratio). A lower ratio is indicative of an increase in anxiety-like behavior [20], [21]. Analysis revealed an overall main effect of genotype [F(1,203) = 12.566, p<0.001] with transgenic mice having a lower ratio relative to wildtype mice (Figure 3c). Unlike other measures of the open field assay, the genotype×age interaction was not significant (p = 0.135).

#### Onset of motor impairments on the rotarod assay occurs early in 110Q mice

**Figure 4:**
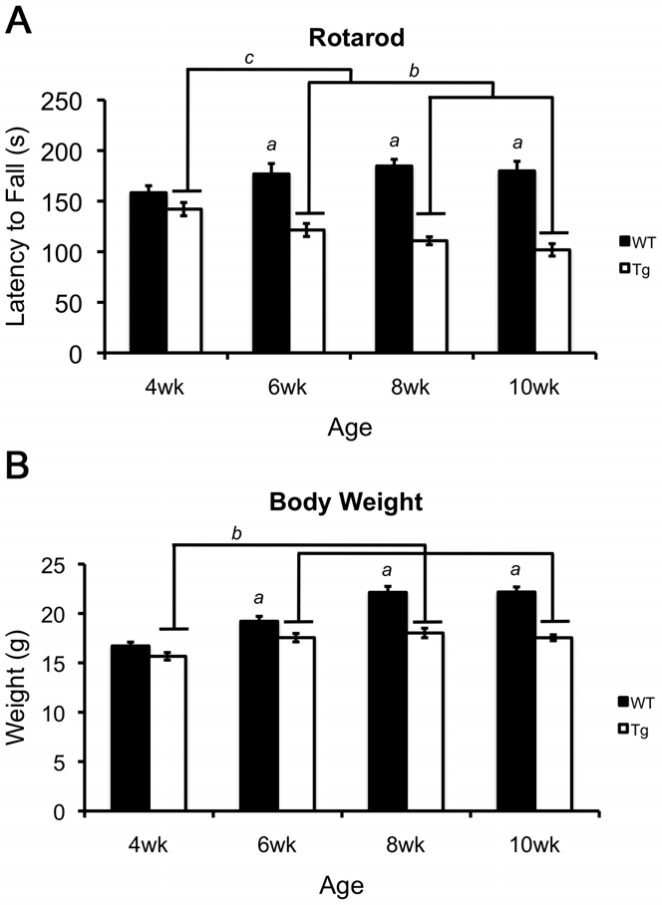
Motor coordination and skill learning on the rotarod and body weights in R6/2 mice across 4 ages. (**A**) Latency to fall from the rotarod. (**B**) Bodyweight measurements from 4 to 10 weeks of age. In both **A** and **B**, ‘*a*’ represents that R6/2 were significantly different from wildtypes. In **A**, ‘*b*’ indicates R6/2 mice at 6 weeks had a longer latency to fall than R6/2 mice at 8 and 10 weeks of age and ‘*c*’ denotes a significant decrease in R6/2 mice at 6, 8 and 10 weeks relative to transgenic mice at 4 weeks old. In **B**, ‘*b*’ denotes an increase in body weight between R6/2 mice at 4 weeks of age and R6/2 mice at 6, 8 and 10 weeks. All p-values≤0.035 by three-way ANOVA and simple effects analysis.

110Q transgenic mice showed motor impairments in the rotarod assay compared to wildtype mice [F(1,204) = 122.994, p<0.001], but this difference was age dependent. Simple effects analysis of the significant genotype×age interaction [F(3,204) = 7.002, p<0.001] showed that as early as 6 weeks of age R6/2 transgenics exhibited impairments in the rotarod assay. A significant decrease in the latency to fall from the rod was observed at 6, 8 and 10 weeks of age in R6/2 mice relative to wildtype mice (p-values<0.001) (Figure 4a). As the mice aged, motor impairments in the R6/2 mice progressed with significant differences between 6 and 10 weeks of age (i.e. 4 weeks>6 weeks>10 weeks; 8 weeks = 6, 10 weeks) (p = 0.039, Table 2). Performance across age in wildtype mice was largely stable across age with a significant increase in performance only occurring between mice at 4 weeks and mice at 8 weeks of age (p = 0.018).

**Table 2:** F-values for interactions found in behavioral tests are shown and simple effects analysis of specific age comparisons within each genotype.

Assay	Interaction	4 wks vs. 6 wks	4 wks vs. 8 wks	4 wks vs. 10 wks	6 wks vs. 8 wks	6 wks vs. 10 wks	8 wks vs. 10 wks
**Total Distance** (genotype×age)	F(1,203) = 16.256, p<0.001	Wt p = 0.573Tg p = 0.215	Wt p = 0.002Tg p = 0.007	Wt p = 0.002Tg p<0.001	Wt p = 0.007Tg p<0.001	Wt p = 0.008Tg p<0.001	Wt p = 0.797Tg p = 0.172
**Vertical Activity** (genotype×age)	F(1,203) = 29.819, p<0.001	Wt p = 0.022Tg p = 0.854	Wt p<0.001Tg p = 0.001	Wt p<0.001Tg p<0.001	Wt p<0.001Tg p = 0.001	Wt p<0.001Tg p = 0.001	Wt p = 0.278Tg p = 0.614
**Rotarod** (genotype×age)	F(3,204) = 7.002, p<0.001	Wt p = 0.132Tg p = 0.039	Wt p = 0.018Tg p = 0.002	Wt p = 0.085Tg p<0.001	Wt p = 0.326Tg p = 0.238	Wt p = 0.813Tg p = 0.039	Wt p = 0.415Tg p = 0.337
**Body Weight** (genotype×age)	F(1,204) = 161.310, p<0.001	Wt p<0.001Tg p<0.001	Wt p<0.001Tg p<0.001	Wt p<0.001Tg p<0.001	Wt p<0.001Tg p = 0.215	Wt p<0.001Tg p = 0.353	Wt p = 0.035Tg p = 0.827
**Prepulse Inhibition**		*Females*					
*Acoustic Startle* (genotype×age×gender)	F(1,203) = 3.273, p = 0.022	Wt p = 0.467Tg p = 0.049	Wt p = 0.263Tg p = 0.040	Wt p = 0.381Tg p = 0.009	Wt p = 0.602Tg p = 0.901	Wt p = 0.860Tg p = 0.455	Wt p = 0.716Tg p = 0.539
		*Males*					
		Wt p = 0.004Tg p = 0.161	Wt p<0.001Tg p<0.001	Wt p<0.001Tg p = 0.003	Wt p<0.001Tg p<0.001	Wt p<0.001Tg p = 0.060	Wt p = 0.460Tg p = 0.226
*Average PPI* (genotype×age)	F(3,203) = 4.569, p = 0.004	Wt p = 0.674Tg p = 0.955	Wt p = 0.636Tg p = 0.914	Wt p = 0.199Tg p = 0.009	Wt p = 0.936Tg p = 0.864	Wt p = 0.081Tg p = 0.008	Wt p = 0.087Tg p = 0.006
**Passive Avoidance** *Days 2 &3* (genotype×age)	F(3,192) = 3.666, p = 0.013	Wt p = 0.011Tg p = 0.311	Wt p = 0.006Tg p = 0.583	Wt p<0.001Tg p = 0.908	Wt p = 0. 438Tg p = 0.125	Wt p = 0.060Tg p = 0.394	Wt p = 0.470Tg p = 0.521

#### R6/2 mice show significant weight loss

110Q mutant mice were found to weigh significantly less than control mice [F(1,204) = 161.31, p<0.001] (Figure 4b). A genotype×age interaction was found [F(1,204) = 161.310, p<0.001]. Specifically, weight differences were apparent by 6 weeks and at later ages of the transgenic mice (p-values<0.001). Although transgenic mice gained weight from 4 to 6 weeks of age (p<0.001), 110Q mice did not continue to gain weight normally after 6 weeks (p-values≥0.215). In contrast, wildtype mice gained weight at every age (p-values≤0.035). Additionally, a genotype×gender interaction was identified [F(1,204) = 10.586, p = 0.001]. Follow-up analyses revealed gender differences in both genotypes (p-values<0.001) with females weighing significantly less than males. Gender differences in body weight are not surprising and not likely to be contributing disproportionately to the overall effect of genotype observed. Furthermore, genotype differences were identified for both genders (p-values<0.001) and both male and female transgenic mice were again shown to weigh significantly less than controls. Although there was a significant gender difference in both genotypes, the significant interaction resulted from a greater mean difference between genders in wildtype relative to R6/2 mice.

#### Transgenic mice show reduced startle and, at later ages, a loss of prepulse inhibition (PPI)

**Figure 5:**
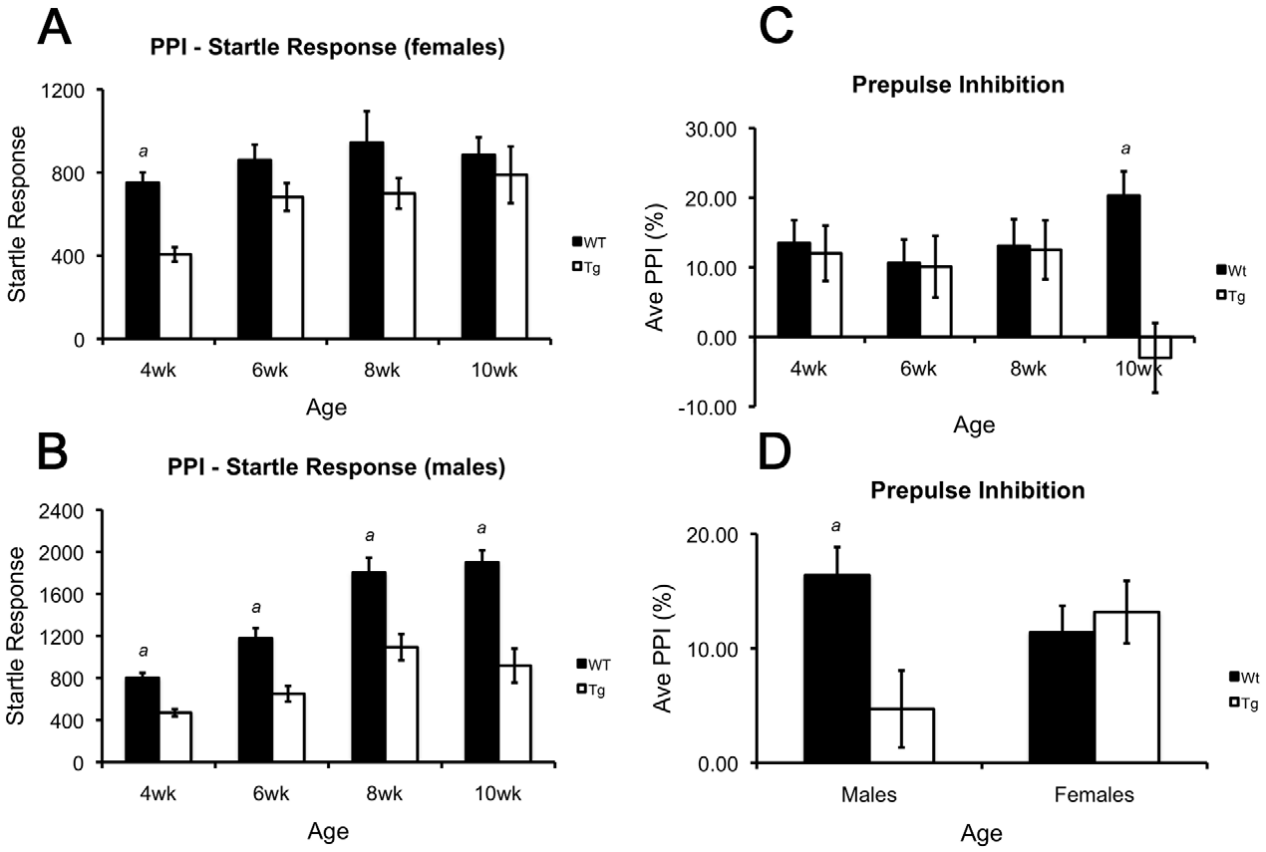
Sensorimotor gating in B6 R6/2 mice across 4 ages. (**A**) Acoustic startle response measurements in male mice. Male R6/2 mice show reduced startle phenotypes at every age tested. The startle response in male R6/2 mice increased significantly from 4 to 6 and 6 to 8 weeks of age, but always remained below wildtype levels. (**B**) Acoustic startle response in female mice. Female R6/2 mice exhibit a significant reduction in startle response only at 4 weeks of age. A significant increase in startle was observed from 4 to 6 weeks in R6/2 females and at all later ages female R6/2 mice showed similar startle responses to wildtype mice. (**C**) Average percent PPI of wildtype and R6/2 mice (combined genders). Average PPI was normal in R6/2 mice at early ages but decreased inhibition was observed at 10 weeks of age. Follow-up analyses revealed that the inhibition deficits were present only in male R6/2 mice. (**D**) Percent PPI in male and female mice. Overall, male R6/2 mice show decreased inhibition compared to wildtype mice while female R6/2 mice performed comparably to controls. In all panels, ‘*a*’ reflects a decreased performance in R6/2 mice compared to wildtypes. All p-values≤0.012 by three-way ANOVA and simple effects analysis.

Overall, the acoustic startle response was reduced in R6/2 mice relative to wildtypes [F(1,203) = 72.451, p<0.001], but this difference was dependent on age and gender as revealed by a genotype×age×gender three-way interaction [F(1,203) = 3.273, p = 0.022]. Both male and female transgenic mice exhibited impairments in the startle response amplitude at 4 weeks old (males: p = 0.012; females: p = 0.025) (Figure 5a–b). Female startle responses increased after 4 weeks of age to wildtype levels (p-values≥0.135, Figure 5a) and although the startle response in male transgenics also increased (from 6 to 8 weeks, p<0.001) they remained significantly below wildtype levels (p-values<0.001, Figure 5b). While no main effects of genotype, age or gender were identified when average PPI values were analyzed, genotype×age [F(3,203) = 4.569, p = 0.004] and genotype×gender interactions [F(1,203) = 6.902, p = 0.009] were found. Further analysis revealed genotype differences only in 10 week old 110Q mice (p<0.001) (Figure 5c), and the difference existed only in male mice (p<0.001) (Figure 5d).

#### HD-model mice have impaired performance in the passive avoidance test

To investigate learning and memory in R6/2 mice we utilized the striatal-based passive-avoidance learning task [22], [23]. Many learning and memory assays require an animal to respond with movement or otherwise perform a task. Due to the motor phenotypes observed in the 110Q R6/2 mice, we reasoned that the passive avoidance task would allow us to identify any motor confounds. Specifically, we believed that if motor impairments interfered with the task they would be easily identified because the mice would take longer to enter the dark thus resulting in a “better” score. For example, if an animal has associated the dark chamber with a foot shock, the animal will avoid the dark chamber, remaining for a longer time in the brightly lit chamber. The longer latency to enter the dark is interpreted as an indication of learning/memory. The higher the latency, the more compelling the data suggesting the animal has learned to associate the dark chamber and foot shock. However, if an animal suffers from severe motor impairments, it may take longer to enter the dark chamber due to the physical limitations thus resulting in a falsely positive learning score for latency to enter the dark. Based on the motor impairments exhibited in R6/2 mice, we would expect the latency to enter the dark during a passive avoidance test to be increased partially due to motor/activity impairments. Therefore, if the R6/2 mice still have lower escape latencies relative to wildtype mice during the memory test then this difference cannot be attributed to impaired activity.

**Figure 6:**
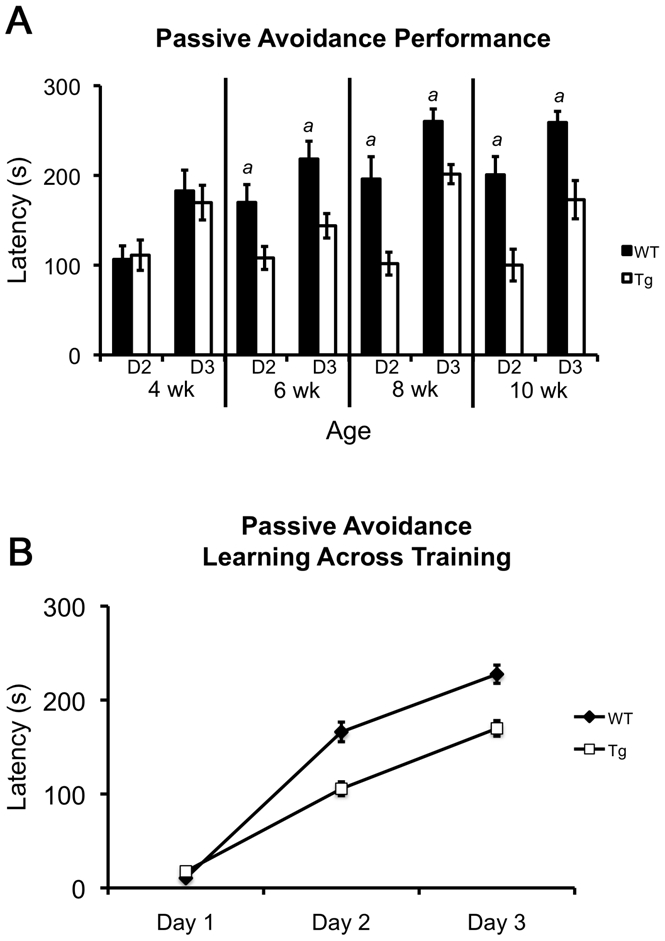
Passive avoidance learning across 4 ages. (**A**) Latency to enter the dark on test days 2 and 3 of passive avoidance test. (**B**) Latency to enter the dark across test days combined across ages. For wildtype and R6/2 mice, latency to enter the dark increased significantly with each training day. R6/2 mice took longer to enter the dark on day 1, but exhibited significantly shorter latencies on days 2 and 3. In panel **A**, ‘*a*’ represents a decreased latency to enter the dark in R6/2 mice compared to wildtypes. All p-values≤0.018 by three-way ANOVA and simple effects analysis.

To look at the performance of naïve mice we analyzed the latency to enter the dark on training day 1 of the assay. Naïve transgenic mice took significantly longer to enter the dark chamber than wildtype mice [Wt: 10.46±0.640, Tg: 17.64±1.143; F(1,192) = 26.672, p<0.001], indicating that indeed they have reduced activity in the passive avoidance assay as was observed in the open field. Data from testing days 2 and 3 revealed an overall effect of genotype [F(1,192) = 31.156, p<0.001] and a genotype×age interaction [F(3,192) = 3.666, p = 0.013]. Further analysis showed that at 4 weeks of age, the latency to enter the dark during test days 2 and 3 in transgenic animals was similar to that of wildtype mice (Wt: 106.44±15.07, Tg: 111.12±16.93; p = 0.801), but by 6 weeks performance of transgenic animals was impaired and the mutant mice entered the dark on days 2 and 3 more quickly than wildtype littermates (Wt: 169.91±19.896, Tg: 108.04±12.817; p<0.001). This impaired performance continued at 8 and 10 weeks of age (8 weeks Wt: 196.0±24.861, Tg: 101.72±12.73, p = 0.018; 10 weeks Wt: 200.7±20.328, Tg: 100.09±17.715, p<0.001) (Figure 6a) but no changes across age were observed in transgenic animals although wildtype mice performed differently at specific ages (Table 2).

In a separate analysis, we asked the question: does the performance of wildtype and transgenic mice improve across days (i.e. are the mice learning)? We found that on day 2 of training both wildtype and transgenic animals exhibited increased latencies to enter the dark compared to day 1 (p-values<0.001) (Figure 6b). In addition, the performance of both genotypes improved again, between days 2 and 3 (p-values<0.001) indicating that although the R6/2 mice are impaired relative to wildtype mice, their performance improves.

### Transgene Expression Levels

Molecular phenotypes have been shown to be associated with disease progression in other HD mouse models [24], [25] and, to the best of our knowledge, no studies have been done in which the molecular phenotypes in the R6/2 model have been studied in association with disease onset or progression. Therefore, in order to more fully characterize the congenic R6/2 mice, we investigated the molecular features of the 110Q transgenic mice. We reasoned that insights into disease onset and progression in the 110Q mice might be gained by understanding the molecular changes that occur in concert with motor and cognitive symptoms.

#### Transgene transcript expression levels do not change with age or disease state in 110Q mice

mRNA from 110Q mice was analyzed at 4, 6, 8 and 10 weeks of age to determine whether changes in gene expression change over time with disease progression. No changes were observed between samples from R6/2 mice at 4, 6, 8 or 10 weeks of age for either transgene (*HTT*) or homologous endogenous gene (*Hdh*) expression (p≥0.205, Table 3).

**Table 3:** Transcript expression across age in 110Q mice.

Age	Gene	Relative Expression (normalized to 110Q at 4 weeks)	SEM^1^	p-value^2^
6 weeks	*HTT*	0.836	0.584–1.289	0.218
	*Hdh*	1.124	0.805–1.465	0.314
8 weeks	*HTT*	0.846	06.31–1.180	0.205
	*Hdh*	0.972	0.794–1.162	0.739
10 weeks	*HTT*	0.881	0.606–1.303	0.341
	*Hdh*	0.926	0.707–1.237	0.452

1 SEM represents the range of standard error of the means for statistical randomization tests.

2 p-values>0.05, *n.s.*

#### Soluble transgene protein levels decrease and aggregate transgene protein levels increase with age and disease progression

**Figure 7:**
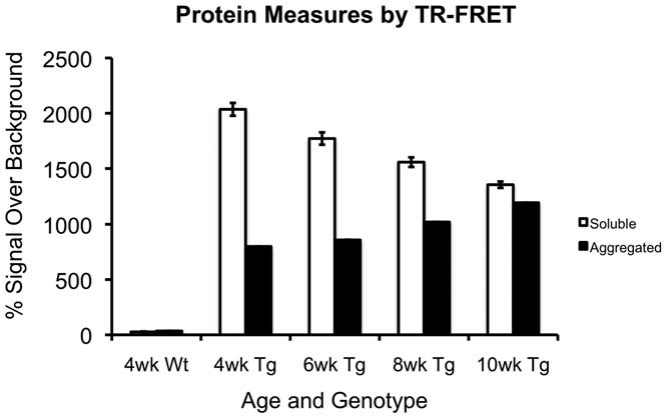
Mutant HTT protein progresses from soluble to aggregated forms with increasing age and disease progression. Relative levels of soluble and aggregated transgene protein across four ages. Soluble protein levels significantly decrease at every age. Aggregated protein levels increase at each age from 6 to 10 weeks. All p-values≤0.018 by one-way ANOVA.

Protein levels from 4, 6, 8 and 10 week old 110Q R6/2 mice showed a pattern of protein state changes in which soluble protein levels decreased with disease onset and progression while aggregated protein levels increased. The 110Q data revealed significant decreases in soluble protein level from 4 weeks of age on (i.e. 4 weeks>6 weeks>8 weeks>10 weeks) (p-values≤0.018) (Figure 7). In addition, time-resolved FRET (TR-FRET) analysis revealed the presence of aggregates in 4 week old 110Q transgenics, prior to phenotypic onset. Although aggregated protein measures from 4 and 6 week old transgenic mice showed no significant increase, levels of aggregated transgene protein rose at each age from 6 to 10 weeks (i.e. 6 weeks<8 weeks<10 weeks) (p-values≤0.001).

### Immunohistochemistry (IHC)

To further confirm the presence of aggregates prior to phenotypic onset as well determine the intracellular localization of aggregates present, the anti-huntingtin antibody, EM48, was used in IHC experiments to look at aggregate load in brain tissue sections of 110Q R6/2 mice.

**Figure 8:**
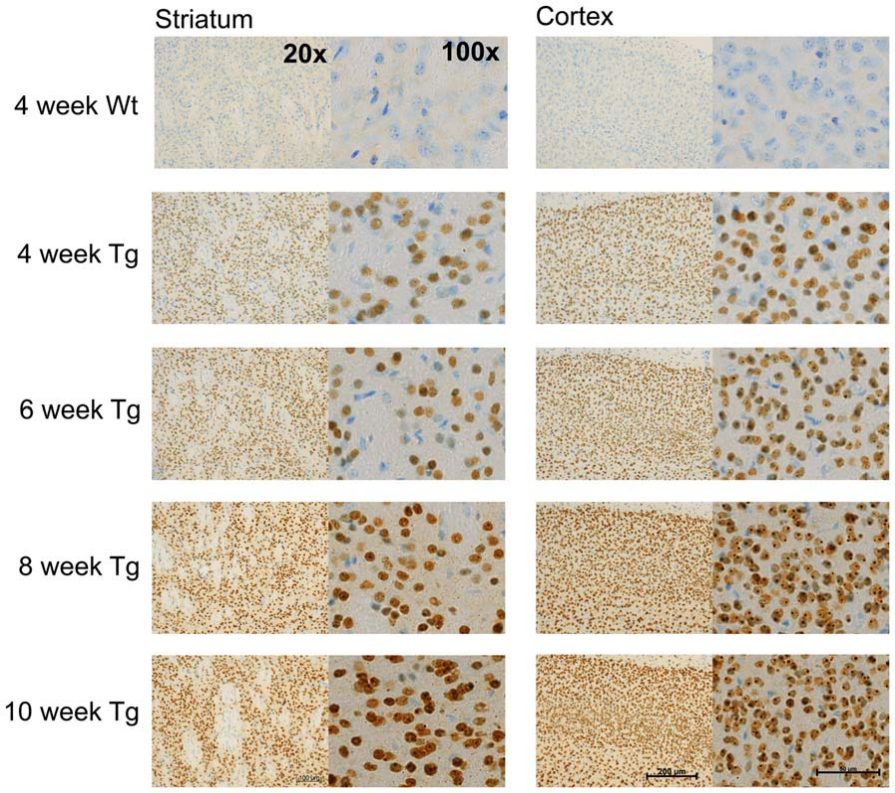
EM48 staining for aggregated mutant HTT appears to increase in amount and intensity with increasing age and disease progression. In 4 week old B6 110Q R6/2 mice, very few small, circular intranuclear inclusions characteristic of 110Q R6/2 mice can be identified. However, by 10 weeks of age such inclusions are readily observed neuronal nuclei. Intensity of EM48 staining appears increase from 4 and 10 weeks of age, further suggesting an increase in aggregate load.

EM48 staining of brain sections from R6/2 mice at 4 and 10 weeks of age again confirmed the aggregate increases reported in the TR-FRET experiments (Figure 8) and showed that aggregates are present prior to explicit phenotype onset (as measured by behavioral testing) in 110Q mice. Although the increase in aggregation from 4 to 10 weeks in the 110Q mice, as seen by IHC is not visually explicit, nonetheless differences are observed. Although not quantified, the EM48-stained sections reveal a clear increase in aggregated HTT protein from 4 to 10 weeks of age in both the cortex and striatum of 110Q mice. In addition tissue samples from 4 week old 110Q mice exhibit few examples of small, circular intranuclear inclusions characteristic of 110Q R6/2 mice and such inclusions are readily identified in the nuclei of neurons of 10 week old 110Q mice. Furthermore EM48 staining appears to darken in intensity between 4 and 10 weeks of age suggesting increased aggregate load. Similar patterns of staining intensity have been observed across age in YAC128 mice [8].

## Discussion

This study reports the generation of congenic R6/2 mice carrying 110 CAG expansion mutation exhibiting minimal instability. By employing a breeding scheme utilizing speed congenics, we illustrate the relative rapidity with which HD-model mice can be backcrossed onto a pure genetic background, allowing for behavioral testing as well as the exploration of differences in HD-like phenotypes on multiple genetic backgrounds and the identification of the potential for modifiers of HD symptoms. Importantly, the discovery of a novel CAG repeat with relative stability while backcrossing our R6/2 colony provided a unique opportunity to study the phenotypes associated with a new line of R6/2 that does not undergo progressive intergenerational expansions and is presented on a congenic background. It is important to note that our intergenerational repeat length stability data are based on the analysis of tissue samples from single ear punches in mice from each generation; within subject somatic repeat stability was not measured in this study. However, because somatic tissue stability has been shown to be separable from traditional intergenerational stability measurements [19], experiments to assess the somatic stability in striatal tissue are currently underway to further characterize the apparent repeat length stability of the 110Q line. At the present time, we do not know why the present 110Q line displays minimal instability, but we speculate that there may be an unidentified mutation within the transgene, or the introduction of an unknown mutation in a modifier locus near the insertion site. Furthermore, although the exact mechanism resulting in expansion of CAG repeats is unknown, many studies have clearly shown a role for mismatch repair proteins in trinucleotide instability [26], [27], [28], [29], [30], [31], [32], [33], [34], suggesting changes in the expression of a protein in the mismatch repair pathway may also explain the relative stability of the 110Q mice.

In the present study, data from congenic 110Q mice exhibiting minimal CAG instability exhibit impairments relative to wildtype mice frequently beginning between 6 and 8 weeks of age. Reduced activity and exploration in multiple measures of the open field assay suggested the onset of motor/exploratory impairments by 6 weeks of age. This result was further confirmed in 6 week old mice when tested in the rotarod assay. Overall, male and female transgenic mice performed similarly to male and female wildtype mice as has been observed in several studies of R6/2 mice [6], [9], [35], [36]. However, gender differences in transgenic phenotypes were observed in the phenotypic development of sensorimotor gating impairments. Deficits in sensorimotor gating have been found among HD patients [37] and have been identified previously in R6/2 mice as well as other animal models of HD [6], [38], [39], [40], [41], [42]. In our study, female R6/2 mice exhibited decreased startle responses at 4 weeks of age while male 110Q R6/2 mice were found to be impaired in startle amplitude at every age as well as in inhibition of the startle at 10 weeks old. Although we identified PPI phenotypes primarily in male transgenic mice, previous studies of R6/2 mice have reported acoustic startle and PPI impairments in both genders [6], [41]. The discrepancy between our data, which showed phenotypes primarily in male transgenic mice, and those of Carter et al. [41] and Menalled et al. [6], which identified phenotypes in both genders, may be explained by differences in ages tested, repeat length, or genetic background, which is known to influence many aspects of behavior [6], [8].

Several studies have shown learning and memory and psychiatric-related phenotypes in R6/2 mice as well as other mouse models of HD including spatial learning, alternation, visual and reversal learning, social interactions and learning and inhibition [35], [36], [38], [40], [43], [44]. In many studies, the results are confounded by motor impairments characteristic of the HD models [5], [40], [41], [42] and very few have successfully separated motor deficits from learning or cognitive impairments [36]. In the present study we have provided evidence in support of a non-motor, striatal-based decreased inhibition phenotype in 110Q R6/2 mice. Because of the nature of the behavioral requirements in the passive avoidance learning and memory task, we were able to show that despite having motor impairments, the R6/2 mice have impaired performance. More specifically, since the motor impairments in R6/2 mice result in reduced activity as seen in their decreased latency on the training day, we expected a longer latency to enter the dark due to the reduced activity on the test days. Yet the data from test days 2 and 3 clearly show that transgenic R6/2 mice entered the dark significantly faster than wildtype mice.

Therefore, despite having motor impairments, the R6/2 mice can engage in the necessary behaviors required to perform the passive avoidance test, and thus the ‘impaired’ performance can be dissociated from a motor impairment. Furthermore, because the transgenic mice improved significantly across days in the passive avoidance task (although performance scores were still significantly below that of wildtype mice), it is clear that the impairments observed are not due to a deficiency in learning or memory *per se*, but is more likely that the decreased latency to enter the dark is the result of problems with inhibition. Our findings contrast to previous findings in which R6/2 mice were found to have a longer latency to enter the dark chamber an inhibitory avoidance task [43]. However, the current study is not the first study to show reduced inhibition, stimulus-response deficits or neuronal dysfunction in the striatum R6/2 mice [36], [43], [45]. For example impairments have been reported in multiple stimulus-response tests including the active avoidance task in which mice must learn to respond to a conditioned stimulus to escape shock [43] and a two-choice water box in which mice are required to swim toward a light to find the escape platform [36]. In addition, Kung et al. [45] illustrate molecular changes in the R6/2 striatum with evidence of impairments in dopamine-dependent neuronal plasticity, providing evidence for striatal dysfunction and a possible mechanism to explain the striatum-based motor and cognitive impairments observed in R6/2 mice. Further studies will be necessary to better understand the nature of the passive avoidance impairment and to more specifically address the possible dissociation between impaired memory and response inhibition.

Furthermore our data suggest that some changes in cognitive or psychiatric-related phenotypes including anxiety in the open field, prepulse inhibition and passive avoidance learning do not precede motor impairments in 110Q R6/2 animals (in which motor impairments occur as early as 4 weeks old). In another study, Lione et al. [36] successfully dissociated cognitive impairments from motor deficits using the Morris water maze. In the Lione study, the decline in performance of transgenic mice in spatial learning occurred before the onset of motor phenotypes. The differential results, with regard to cognitive or psychiatric features occurring prior to motor phenotypes, between our study and Lione et al. [36] may reflect different types of learning, differentially affected brain regions (i.e. the striatum or the hippocampus), or be due to the differences in genetic background or repeat length, although the latter was not reported.

In addition to characterizing the behavioral phenotypes of 110Q congenic R6/2 mice with minimal mutation instability, the findings of the present study expand the current knowledge of R6/2 mice by elucidating several molecular changes associated with disease onset and progression in the R6/2 model. Among the molecular phenotypes associated with disease, changes in the levels of soluble and insoluble mutant HTT protein (as measured by TR-FRET) were found. We observed a decrease in soluble protein with disease onset and a concurrent increase in aggregated protein levels. However, we found no differences in transgene transcript expression in R6/2 mice; that is, transcript expression levels were stable throughout disease progression and were not associated with disease onset. This suggests that protein state changes, and not changes in transcript expression, may be associated with disease onset and progression.

At later ages (i.e. 8–10 weeks), data from the present study show that the congenic B6 110Q mice that show intergenerational stability parallel many of the phenotypes commonly observed in R6/2 mice on a mixed genetic background that do not show intergenerational stability including reduced body weight, motor impairments and decreased exploration [5], [6], [9], [35], [36], [41]. Although 110Q R6/2 mice were largely healthy and perform comparably to wildtype littermates at 4 weeks of age, impairments in startle and sensorimotor gating were observed at this young age suggesting that some aspects of disease occur early in development in R6/2 mice. In addition, the present study provides evidence for striatal-dependent inhibition impairments in a learning and memory assay [22], [23] in B6 110Q R6/2 mice that are not present prior to the onset of HD-like motor symptoms. Finally, we show a progression of disease onset in B6 110Q mice across age both behaviorally and in molecular changes in protein state and aggregation of the mutant transgene product. While previous studies have shown similarly progressive phenotypes in both the R6/2 and other HD mouse models [8], [35], [36], [41], [42], [46], no concurrent progressive molecular phenotypes have been published on R6/2 mice. Our results, however, are neither surprising nor unique to the R6/2 model. YAC128 mice have been reported to exhibit correlations between behavioral disease progression accompany progressive changes at the molecular level including susceptibility to excitotoxicity and cortico-striatal activity [24], [25]. Taken together, the results of our study and those observed in YAC128 mice suggest that insights into disease onset, mechanism, and progression can be gained through understanding the molecular changes that occur in concert with motor and cognitive symptoms.

## Materials and Methods

### Ethics Statement

Animal care and testing was approved by the Baylor College of Medicine Animal Care and Use Committee under protocol number AN-1695 and was performed in accordance with NIH guidelines.

### Animals

Male R62 mice carrying a transgene with a 110-polyglutamine (110Q) tract (Jackson Laboratory, Bar Harbor, ME) were originally obtained on a mixed CBA×C57BL/6J background. Using speed congenics mice were backcrossed onto C57BL/6J (B6) background based on a 96 short sequence length polymorphisms (SSLPs) marker panel until congenic (details provided in Marker-Assisted Speed Congenics methods section). After obtaining a fully B6 congenic 110Q R6/2 line, female B6 110Q R6/2 mice underwent ovarian transfer (OT) from a transgenic to a wildtype female at Jackson Laboratory. The female OT R6/2 mice were then returned to our lab at Baylor College of Medicine and were bred to male B6 mice in our colony. Weaned progeny were housed 2–5 animals per cage with a 12-hour light cycle. Food and water were provided ad libitum.

### Marker-Assisted Speed Congenics

In order to efficiently create a congenic B6 R62 line, a marker-assisted speed congenics selective breeding strategy was used [47], [48]. A 96 SSLP marker panel was chosen using Jackson Laboratories online genes and markers database (http://www.informatics.jax.org/genes.shtml). Approximately 4–5 markers were chosen on each chromosome with no more than 20 cM between markers to enhance detection of recombination events. PCRs assaying all 96 markers were performed for each mouse, and PCR products were analyzed on poly-acrylamide gels. Male 110Q mice from each generation containing the highest percentage of homozygous B6 markers were used for breeding. Mice were backcrossed until all 96-markers analyzed were found to be homozygous B6.

### Genotyping and CAG Repeat Length

All mice were genotyped using PCR amplification of DNA extracted from tails clippings. Transgenic animals were identified using primers (5′-GCCGCTCAGGTTCTGCTTT-3′ and 5′-AAGGCCTTCATCAGCTTTTCC-3′) to amplify the 5′ region of the transgene, yielding a 150 base-pair product. Tissue obtained from ear clips of all mice were sent to Laragen, Inc. (Los Angeles, CA) to measure the polyglutamine expansion by Genescan and sequencing modes using an ABI 377 sequencer. Mice used in this study had repeats ranging from 107–115 CAG.

### Insertion Site Characterization

Genomic sequences flanking the transgene integration site were identified using the PCR-based Genomewalker Universal Kit (Clontech Laboratories, Inc., Mountain View, CA) according to the manufacturer's instructions. In summary, restriction enzyme digestions were performed using purified high molecular weight genomic DNA isolated from R6/2 and wild-type mice. Kit supplied GenomeWalker Adaptor oligos were ligated to the genomic DNA fragments and primary PCR amplification was carried using a gene-specific primer (GSP1; 5′-AGGACAAGGGAAGACCCAAGT-3′) and an Adaptor-specific primer (AP1; see manufacturer protocol). A second, nested PCR amplification was carried out using diluted primary PCR product as template and a second gene-specific (GSP2; 5′-TCTGGGTTGCTGGGTCACTCT-3′) and Adaptor-specific primer (AP2; see manufacturer protocol) pair. The final amplification products were resolved on an agarose gel and visible band(s) were isolated, purified, and then sequenced. Publicly available homology search programs were then utilized to identify the chromosomal region flanking the construct (http://genome.ucsc.edu/cgi-bin/hgBlat). The transgene insertion site was further confirmed by follow-up targeted PCR using primers 5′GCAAGCAAGGGGAGGAAAAG-3′ and 5′-AGGCCCATGCGGAAAGGAT-3′.

### Behavioral Testing

Male and female 110Q mice were tested in a variety of behaviors in the following order: (1) open-field activity, (2) rotarod, (3) prepulse inhibition and (4) passive avoidance. Mice were used in one test per day and each test was performed with no less than one and no more than three days in between. In general, testing was completed over a ten-day period. Mice were given a 30-minute rest period after being moved to testing rooms each day. All behavioral experiments were carried out between 8 AM and 1 PM. N for each age group tested were as follows: 4 week n = 13 Tg females, n = 14 Tg males, n = 10 Wt females, n = 17 Wt males; 6 week n = 14 Tg females, n = 19 Tg males, n = 14 Wt females, n = 14 Wt males; 8 week n = 14 Tg females, n = 17 Wt males, n = 8 Wt females, n = 15 Wt males; 10 week n = 13 Tg females, n = 10 Tg males, n = 13 Wt females, n = 14 Wt males. Naïve mice were used in each age group. The average repeat length for mice used in behavioral experiments is 112.48±1.082 SEM.

#### Open Field Activity

Mice were transferred from a cage to the center of a clear Plexiglas arena (40×40×30 cm). The duration of the test was 30 minutes, during which time mice were allowed to explore freely. Overhead lighting of 800 Lux was placed above the open field chamber. White noise was provided at 55 dB during testing. The VersaMax Animal Activity Monitoring System (AccuScan Instruments, Inc., Columbus, OH) was used to monitor and record activity during the test. Activity was detected by photobeam interruptions and measures including total distance traveled, rearing and distance traveled in the center of the arena (22.5 cm×22.5 cm). The ratio of the center distance to the total distance (center∶ total distance ratio) was also used as an index for anxiety-like behaviors [20], [21]. Beam breaks were computer recorded over 2-minute intervals throughout the test period. Data from the entire 30-minute test were analyzed.

#### Rotarod

Mice were placed on an UGO Basile Accelerating Rota-Rod (Ugo Basile Company, Collegeville, PA) and the latency to fall was recorded as a measure of motor skill and learning. When an animal lost its balance but did not fall, and continued to hold on to the rod, the time to the first full revolution was recorded. Acceleration of the rod was from 4 to 40 rpm over a 5-minute trial period. Mice were given four trials per day, with an intertrial interval of 20–40 minutes, over two consecutive days. The latency was averaged across all eight trials and analyzed. Prior to testing on day one, the weight of each animal was measured and recorded.

#### Prepulse Inhibition (PPI)

Acoustic startle responses were measured as to indicate sensorimotor gating performance using the SR-Lab System (San Diego Instruments, San Diego, CA). Mice were restricted inside holding tubes and presented with a 5 minute 70 dB background noise. In a single test session, six blocks of trials were administered pseudo randomly. Each block consisted of eight trial-types each trial-type was presented once within each block; intertrial intervals ranged from 10–20 s. “Startle only” trials consisted of a 40 ms, 120 dB sound. In three trials-types, prepulses consisting of 4, 8 or 12 dB above the 70 dB background were presented. Another three trials both prepulse and acoustic startle stimulus. In the prepulse+startle trials, prepulses were presented for 20 ms, 100 ms prior to the startle. In the last trial-type only background noise was presented (for measuring baseline movement). Response detection occurred for 65 ms following stimulus presentation. Maximum startle amplitude was used as the dependent variable and mice with startle amplitude less than 100 were excluded from study results.

Percent prepulse inhibition of a startle response for each prepulse level was calculated as follows: 100−[(startle response on acoustic prepulse+startle stimulus trials/startle response alone trials)×100]. The average PPI of all three prepulse intensities was used as the measure for this experiment.

#### Passive Avoidance (PA)

The three-day passive avoidance test was conducted in a two-chamber box (42×16×21 cm) (Med Associates, Saint Albans, VT, USA) divided by a white partition containing a sliding door. One chamber is clear and brightly lit (approximately 800 Lux) while the other is covered and kept dark. Each day mice were placed in the light side of the box and the partition was opened after 10 s. The latency to enter the dark side was recorded. After a mouse entered the dark, the partition door was closed and a 2 s, 0.5 mA foot shock was administered via a grid floor; after 10 sec the mouse was returned to its home cage. Approximately 24-hours later, mice were put back into the light compartment with the partition open and the latency to enter the dark was recorded with a maximum of 300 s. The protocol was repeated approximately 24 hours later. After delivery of the shock, vocalizations were noted, as an indication of the animal detected the footshock stimulus. The latency to enter the dark on day three was used as the measure of learning in this experiment.

#### Statistical Analyses for Behavior

Comparisons were made between genotypes independently at each age. Data for OFA, RROD, and PPI and PA day 1 were analyzed using three-way (genotype×age×gender) Analysis of Variance (ANOVA). When interactions were identified, further statistical analyses were performed using simple-effects tests. Genotype×age analyses revealed changes across age in some assays. The full details of these progressive phenotypes are given in Table 2. Gender differences were identified in weight and in PPI startle responses. The gender×genotype interaction found in the weight data was due to a greater mean difference between genders in wildtype relative to R6/2 mice. Because this was not crucial to the interpretation of the weight measures, the data from both genders were collapsed for the data presented in the figure. Data from PPI startle testing is shown separately for each gender. All other figures represent combined data from both genders. Passive avoidance results for days 2 and 3 were analyzed using a 3-way ANOVA with repeated measures. All three days were analyzed independently for each genotype using 1-way ANOVA with repeated measures to look at performance changes across day.

### Transgene Expression Analysis

Mice used in qPCR and TR-FRET experiments expressed a mutant transgene with an average of 110 CAG (109.73±0.248 SEM).

#### Tissue Collection

Upon completion of behavior studies, mice were sacrificed by cervical dislocation and whole brain tissue was immediately removed and stored in Ambion RNA*later* tissue collection RNA stabilization solution (Applied Biosystems/Ambion, Austin, TX) at 4°C for 1–7 days after which tissue was removed from excess RNA*later* solution and stored at −80°C.

#### RNA Isolation and cDNA Synthesis

Total RNA was isolated from mouse whole brain tissue and dounce homogenized in Ambion TRI-Reagent. Phase separation of homogenate was performed according to the manufacturer's protocol by addition of bromo-chloro-propane, incubation, and centrifugation. The aqueous RNA phase was transferred, treated with ethanol and column purified using the Ambion RiboPure Kit. Genomic DNA was removed by treatment with Ambion TurboDNAseI (Applied Biosystems/Ambion). Quality of RNA was determined by nanodrop UV absorbance and with the Agilent 2100 Bioanalyzer. cDNA was generated using approximately 1 µg of RNA and SuperScriptIII Reverse Transcriptase (Invitrogen, Carlsbad, CA) and poly-T plus random hexamer primers. Following cDNA synthesis, excess RNA was eliminated using RNAseH enzyme (Invitrogen). cDNA was stored at −20°C.

#### geNorm Analysis and qPCR

To determine the reference genes that are most stable in whole brain tissue across disease state and age, total RNA from 4 each of 4, 6, 8 and 10 week old mice were used to perform a geNorm analysis using seven commonly used reference genes. For each gene, the primer and probe used were developed as a Taqman gene expression assay (Applied Biosystems, Foster City, CA) or a primer and double-dye (Taqman-style) assay (PrimerDesign, Ltd., UK). Series dilutions were run for each gene assay and efficiencies were calculated using linear regression analysis. qPCR was performed and results were analyzed using geNorm software (http://medgen.ugent.be/~jvdesomp/genorm/). The three most stable reference genes were selected for use in gene expression experiments.

Following geNorm analysis, gene expression experiments were performed using reference genes CanX (Applied Biosystems, Mm00626988_m1), Cyc1 (Applied Biosystems, Mm00470541_g1) and Rpl13a (Applied Biosystems, Mm01612986_gH). The primers used for amplifying the R62 transgene (HTT) were 5′-GCTGCACCGACCGTGAGT-3′ and 5′-CGCAGGCTGCAGGGTTAC-3′ and a Taqman probe 6-FAM- CAGCTCCCTGTCCCGGCGG-TAMRA. Expression of the endogenous mouse homolog, Hdh, was also measured using qPCR with primers 5′-CTCAGAAGTGCAGGCCTTACCT-3′ and 5′-GATTCCTCCGGTCTTTTGCTT-3′ and Taqman probe 6-FAM-TGAATCTTCTTCCATGCCTGACCCGA-TAMRA. The efficiency for each gene assay is as follows, HTT (78.2%), Hdh (99.8%), Canx (94.97%), Cyc1 (96.7%), and Rpl13a (100.0%). 8 biological samples each of 4, 6, 8 and 10-week old mice were used to measure expression levels.

### Statistical Analyses for Real Time RT-qPCR

REST 2009 Software program [49] was used to analyze data from each experiment. Relative expression was calculated by dividing the concentration of the gene of interest by the geometric mean of the concentrations of reference genes used in each study [50]. Simple statistical randomization tests were run with 10,000 iterations to determine expression ratios [51]. A hypothesis test and bootstrapping methods were then used to determine significance at the 95% confidence interval.

### Protein Isolation and Time-Resolved FRET Analysis

Crude brain tissue homogenates from 4 wk Tg (n = 9), 6 wk Tg (n = 7), 8 wk Tg (n = 8), 10 wk (n = 8) and 4 wk Wt (n = 8) were prepared by homogenization in 10× w/v lysis buffer (PBS+1% TritonX100+Complete Protease Inhibitor (Roche, Switzerland)) by ultrasound pulses. 5 µl homogenate and 1 µl detection buffer (50 mM NaH_2_PO_4_, 400 mM NaF, 0.1% BSA and 0.05% Tween+antibody mix) were pipetted to low-volume wells of opaque white 384 microtiter plates (Greiner, USA). The final antibody amount per well was 1 ng 2B7-Tb+10 ng MW1-d2 or 1 ng 4C9-Tb+10 ng 4C9-Alexa488 for the quantification of soluble mutant huntingtin and aggregated mutant Huntingtin respectively. Plates were incubated for 1 h at room temperature and samples were analyzed with an EnVision Reader (Perkin Elmer, USA). The donor fluorophore terbium was excited at 320 nM. After a time delay of 100 µs, terbium, d2 and Alexa488 emission signals were read out for 200 µs at 620 nm, 665 nm and 520 nm respectively. Data are presented as percentage signal over lysis buffer background.

For TR-FRET data, independent comparisons were made across age for soluble and aggregated protein measures using one-way ANOVA.

### Immunohistochemistry

Mice were sacrificed by transcardial perfusion with 30–40 ml of filtered-sterilized 0.2 M PBS, pH 7.2. Tissues were then fixed by perfusion with 50 ml of cold 4% paraformaldehyde in 4% sucrose PBS, pH 7.2. Whole brains were dissected and stored overnight in the fix solution at 4°C. Within 24 hours post-perfusion, brain samples were transferred to a filter-sterilized 0.2 M PBS containing 0.01% sodium azide and kept at 4°. Brain samples were paraffin embedded and coronally sectioned by Neuroscience Associates, Inc. (Knoxville, TN) into 35-micron serial sections. Immunolabeling with EM48 and thionine counterstain was performed at 210-micron intervals. For this analysis, 4 wk Wt n = 4, 4 wk Tg n = 6, 6 wk Tg n = 5, 8 wk Tg n = 5 and 10 wk Tg n = 5. For this study, the expansion size again showed minimal instability with an average CAG number of 110 (110.35±0.357 SEM).
